# Bioinformatics analysis identified apolipoprotein E as a hub gene regulating neuroinflammation in macrophages and microglia following spinal cord injury

**DOI:** 10.3389/fimmu.2022.964138

**Published:** 2022-08-24

**Authors:** Xin-Qiang Yao, Jia-Ying Chen, Zi-Han Yu, Zu-Cheng Huang, Regan Hamel, Yong-Qiang Zeng, Zhi-Ping Huang, Ke-Wu Tu, Jun-Hao Liu, Yan-Meng Lu, Zhi-Tao Zhou, Stefano Pluchino, Qing-An Zhu, Jian-Ting Chen

**Affiliations:** ^1^ Division of Spine Surgery, Department of Orthopaedics, Nanfang Hospital, Southern Medical University, Guangzhou, China; ^2^ Department of Comprehensive Medical Treatment Ward, Nanfang Hospital, Southern Medical University, Guangzhou, China; ^3^ Department of Clinical Neurosciences, University of Cambridge, Cambridge, United Kingdom; ^4^ Division of Spine Surgery, Department of Orthopaedics, Guangzhou First People’s Hospital, School of Medicine, South China University of Technology, Guangzhou, China; ^5^ Center of Electron Microscopy, Central Laboratory, Southern Medical University, Guangzhou, China

**Keywords:** apolipoprotein E, neuroinflammation, macrophages, microglia, spinal cord injury, bioinformatics analysis

## Abstract

Macrophages and microglia play important roles in chronic neuroinflammation following spinal cord injury (SCI). Although macrophages and microglia have similar functions, their phagocytic and homeostatic abilities differ. It is difficult to distinguish between these two populations *in vivo*, but single-cell analysis can improve our understanding of their identity and heterogeneity. We conducted bioinformatics analysis of the single-cell RNA sequencing dataset GSE159638, identifying apolipoprotein E (APOE) as a hub gene in both macrophages and microglia in the subacute and chronic phases of SCI. We then validated these transcriptomic changes in a mouse model of cervical spinal cord hemi-contusion and observed myelin uptake, lipid droplets, and lysosome accumulation in macrophages and microglia following SCI. Finally, we observed that knocking out APOE aggravated neurological dysfunction, increased neuroinflammation, and exacerbated the loss of white matter. Targeting APOE and the related cholesterol efflux represents a promising strategy for reducing neuroinflammation and promoting recovery following SCI.

## Introduction

Traumatic spinal cord injury (SCI) can lead to permanent neurological disorders (1). The dynamic SCI environment has three phases: the acute phase, occurring up to three days post injury (dpi), is characterized by hemorrhage, cell death, and cytokine release; the subacute phase, occurring 3-14 dpi, is characterized by angiogenesis, immune cell infiltration, and phagocytosis of myelin debris; and the chronic phase, occurring more than 14 dpi, is characterized by glial scar formation, remyelination, and neural remodeling (2). Persistent inflammatory processes and neuronal damage are associated with failed functional recovery. Macrophages are mainly immersed in the injury core and are associated with both pro- and anti-inflammatory effects, whereas microglia are mainly located in the injury rim and are associated with a pro-inflammatory role (3). Macrophages and microglia act as ‘‘professional phagocytes’’ following SCI (4). Although macrophages have stronger phagocytic activity, they are less efficient at processing phagocytosed material (5). Therefore, a better understanding of the mechanisms underlying processes such as microglial uptake and digestion is essential. Unfortunately, distinguishing macrophages from microglia is difficult (5).

Single-cell RNA sequencing (scRNA-seq) has facilitated the study of macrophage and microglial complexity following SCI (6). scRNA-seq analysis has been conducted for almost all cell types involved in angiogenesis, gliosis, and fibrosis in a mouse model of SCI (7). Macrophages and microglia are divided into different transcriptional profiles and subpopulations, depending on their functions and tasks (8). A disease-associated microglial subtype was potentially protective against wound healing following SCI (9). FABP5+ macrophages and microglia are regarded as proinflammatory myeloid cells with neurotoxic effects (10). A previous proteomic analysis of a rat SCI model showed that the protein cluster continuously upregulated in the acute and subacute phases was enriched in markers of myeloid cells, lipid regulation pathways, and lysosomes (11). Lipid droplet formation occurs in macrophages and requires lysosomal degradation following SCI (5, 12). Excessive lipid levels lead to the formation of foam cells, which have pro-inflammatory effects (13, 14). Macrophages are more prone to cell death than microglia during the phagocytic response that occurs following SCI (15). The phagocytic mechanism in macrophages and microglia is still not fully understood, and single-cell analysis may help us differentiate these cells (16).

In this study, we analyzed the scRNA-seq dataset GSE159638 to explore differentially expressed genes (DEGs) and pathways that are activated in macrophages and microglia during the subacute and chronic phases of SCI. We then employed histological analysis of a mouse model of cervical spinal cord hemi-contusion to experimentally validate the results of the bioinformatics analysis. Finally, we analyzed the effect of apolipoprotein E (APOE) knocked out on neurological function.

## Materials and methods

### Data acquisition

The GSE159638 count matrices were downloaded from the Gene Expression Omnibus (GEO) database (https://www.ncbi.nlm.nih.gov/geo/). The dataset contains 25 spinal cord samples; five in the one dpi group, four in the two dpi group, seven in the three dpi group, five in the 10 dpi group, two in the 21 dpi group, and two in the sham group. The dataset contains a total of 30,958 cells. The 10X Genomics Chromium™ 3’ single cell solution was used as the single-cell capture platform while GPL24247 (Illumina NovaSeq 6000, Mus musculus) was used as the sequencing platform. Each sample contained either resident microglia or infiltrating myeloid cells, including infiltrating macrophages. These populations were isolated *via* FACS, based on fate mapping labels described in the original article (10).

### Data processing

Downstream data analysis was performed using the “Seurat” package, version 4.10, in R version 4.1.2 (17). Cells containing more than 15% mitochondrial genes were filtered out and the remaining data was normalized using the “LogNormalize” function. Dimensionality reduction was conducted using “FindVariableFeatures” and “RunPCA,” functions and visualized using uniform manifold approximation and projection (UMAP) analysis. Clusters were identified using the following settings: FindNeighbors (dims=1:13), FindClusters (resolution=0.5), and RunUMAP (reduction = “pca,” dims = 1:13). Clusters with fewer and dispersed cells were excluded from further analyses. Cell marker genes and four myeloid cell subtypes were identified using “FindAllMarkers (min. pct = 0.25, logfc.threshold= 0.25)”.

### Group-specific macrophage and microglia markers

A standard area under the curve (AUC) classifier was used to identify macrophage and microglia gene markers expressed at different time points using “FindAllMarkers (min.pct = 0.1, logfc.threshold = 0.25)”. Gene Ontology (GO) analysis of marker genes was performed using the “clusterProfiler” package, version 4.2.2 (18), and three GO terms representing the main functions at each time point were selected from the top 30 terms.

### Differential gene expression analysis

Since macrophages were not detected in the sham group, we used the mean value of macrophage gene expression for the first three days to represent the transcriptional profile of the acute phase. Genes expressed by macrophages 10 and 21 dpi were compared with those expressed over the first three days using the “FindAllMarkers (logfc.threshold=0.5, p_val_adj=0.05)” function. DEGs in microglia were identified by comparing each experimental group with the sham group. GO analysis of overlapping genes expressed 10 and 21 dpi was performed in macrophages and microglia.

### Identification of time-dependent gene expression modules

Macrophage and microglia modules with consistent expression patterns at different time points were categorized using fuzzy c-mean clustering implemented in the “Mfuzz” package, version 2.54.0 (19). First, the average expression of each gene at each time point was calculated for macrophages and microglia. An analysis workflow based on “filter.std (min.std = 0)”, “standardise ()”, and “mestimate ()” functions was then used. Third, clusters containing upregulated genes at 10 and 21 dpi were analyzed against c2 Kyoto Encyclopedia of Genes and Genomes (KEGG) gene sets using gene set variation analysis (GSVA) implemented using the “GSVA” package (version 1.42.0) and “Msigdbr” package (version 7.4.1). Finally, the gene clusters were compared with the DEGs, and genes overlapping the two datasets were subjected to protein-protein interaction (PPI) network analysis using STRING software (20).

### Animal experiments

The animal experiments were approved by the Laboratory Animal Care and Use Committee of Nanfang Hospital, Southern Medical University, and performed according to the National Guidelines for the Care and Use of Animals. Eight-week-old wild-type and APOE^-/-^ male mice were obtained from the Laboratory Animal Center of Southern Medical University and housed at the Laboratory Animal Center of Nanfang Hospital with ad libitum access to food and water.

### Cervical spinal cord hemi-contusion injury

The mice were anesthetized using isoflurane (3% for induction and 1.5%–2% for maintenance) and C5 hemi-contusion injuries were induced as previously described (21). Briefly, the C5 lamina was exposed and removed. A contusion SCI was induced at C5 using an impactor tip (diameter=1.0 mm) with a preset displacement of 1.2 mm at 300 mm/s, controlled by an electromagnetic servo material testing machine (Instron E1000, Instron, United States). Only C5 laminectomy was performed as part of the sham surgery.

### Behavioral assessment

Behavioral assessments were performed as previously described (22). Behavioral assessment was conducted by two independent researchers at different time points before and after surgery. Cylinder rearing test was used to evaluate the use of forelimbs (23). Mouse activity in a 20 cm-diameter transparent cylinder was recorded for 15 min and the first 20 climbing movements (left forelimb touch, right forelimb touch, and both forelimbs touch) or all climbing movements within 15 min were recorded. The grooming test is mainly used to evaluate the motion of the shoulder and elbow joints, with scores ranging from 1 to 5 based on the position of the foreclaws contacting the head and face (24).

### Immunofluorescence staining

Animals were anesthetized with sodium pentobarbital and perfused transcardially with phosphate-buffered saline (PBS), followed by 4% paraformaldehyde. Spinal cords (5 mm rostral to 5 mm caudal to the epicenter) were dissected, postfixed overnight with paraformaldehyde, and dehydrated in 12%, 18%, and 24% sucrose solutions at 4°C. The samples were embedded in optimal cutting temperature compound (Tissue-Tek, 4583, Sakura), and sliced transversely into 20 µm sections using a Leica CM1950 cryostat. For myelin basic protein (MBP) staining, slices were washed once with PBS and then permeabilized in a graded series of ethanol solutions (50%, 70%, 90%, 95%, 100%, 100%, 95%, 90%, 70%, and 50%). The slices were blocked with 0.01 M PBS containing 0.1% Triton X-100 and 10% normal donkey serum for 30 min. The slices were then incubated overnight at room temperature with the following primary antibodies: anti-F4/80 (1:200, Cell Signaling Technology 71299S), anti-APOE (1:400, Abcam ab183597), anti-CD68 (1:400, Abcam ab125212), anti-GFAP (1:800, Cell Signaling Technology 3670S), anti-MBP (1:400, Abcam, ab4039), anti-SMI312 (1:800, Covance, SMI-312R-100), and anti-LAMP2 (1:200, Abcam, ab13524). The following day, the slices were washed three times in PBS and incubated at room temperature for two hours with the following secondary antibodies: donkey anti-mouse Alexa Fluor 488 (1:200, Abcam ab150105), donkey anti-rabbit Alexa Fluor 555 (1:200, Abcam ab150062), and donkey anti-rat Alexa Fluor 647 (1:200, Abcam ab150155). To stain lipid droplets, the slices were incubated with BODIPY (1:400, Invitrogen D3922) for 20 min following their incubation with primary antibodies. They were then washed thrice in PBS and mounted with Fluoromount-G (0100-20, Southern Biotech). Images were captured and analyzed using a Zeiss confocal microscope (LSM980, Zeiss, Germany) and ZEISS ZEN 3.3 software.

### Transmission electron microscopy (TEM)

The mice were transcardially perfused with PBS and 4% paraformaldehyde, and the injury epicenters harvested, postfixed in 2% glutaraldehyde, and incubated overnight at 4°C. The samples were then rinsed in PBS, incubated with osmium tetroxide for 1 h, rinsed in PBS, dehydrated in 30%, 50%, 70%, 90%, and 100% ethanol solutions, and permeabilized in a graded series of acetone-Epon mixtures (1:1 for 1 h, 1:2 for 2 h, 1:2 for 3 h, and pure Epon overnight). Spinal cords were embedded in Epon, sliced into 0.8 µm semi-thin and 60–90 nm ultrathin sections using ultramicrotome Leica UC7. The sections were subsequently stained with uranyl acetate and lead citrate and analyzed under a transmission electron microscope (HITACHI H-7500, Japan).

### Statistical analyses

Statistical analyses were performed using GraphPad Prism (version 8.2.1). Unpaired *t*-test was used to analyze injury-related biomechanical parameters of wild-type and APOE^-/-^ mice. Behavioral assessments and weight changes were investigated using two-way ANOVA with repeated measurement, followed by Bonferroni multiple comparison test between groups at each time point. Kruskal-Wallis test followed by Dunn’s test *post hoc* was used to analyze the immunofluorescence results of F4/80, APOE and BODIPY at different time point. Mann-Whitney test was used to analyze the immunofluorescence results of GFAP, CD68, F4/80, MBP and SMI312 between wild-type and APOE^-/-^ mice. Statistical significance was set at *p*< 0.05.

## Results

### Identification of macrophage and microglia clusters

**Figure 1:**
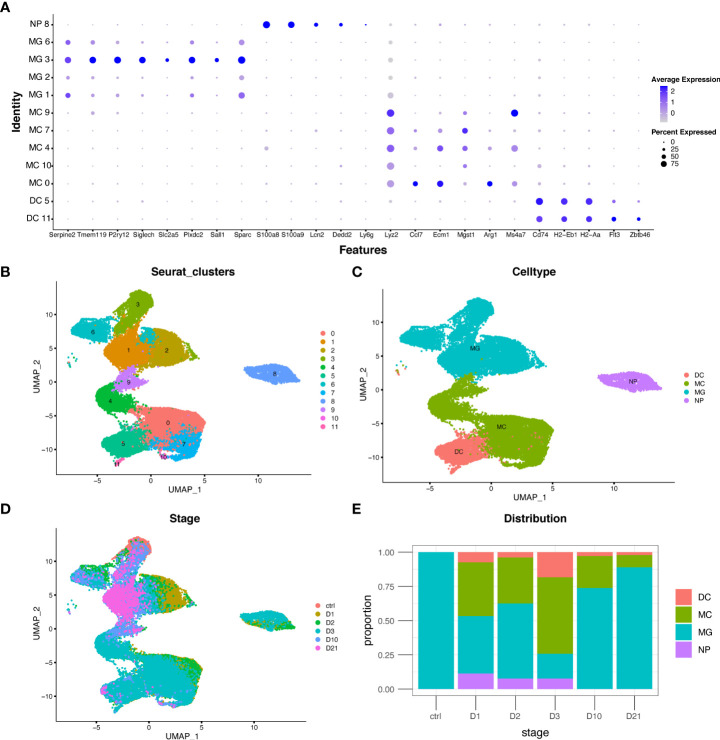
Distribution and proportion of myeloid cell subtypes following spinal cord injury (SCI). **(A)** Dot plot of marker genes in each population. **(B–D)** Uniform manifold approximation and projection (UMAP) plot of each population, four myeloid cell subtypes, and cells from different time points. **(E)** Bar graph showing the proportion of myeloid cell subtypes from different time points. DC, dendritic cells; MC, macrophages; MG, microglia; NP, neutrophils.

The GSE159638 dataset introduced a mouse spinal contusion model using an impactor tip (1.3 mm) and an impact of 70 kilodyne force. Transcriptional profiles of 30,768 cells and 15,731 genes were obtained after quality control. The UMAP algorithm was used to convert the multidimensional data into a two-dimensional plot (**Figure 1B**). Four myeloid cell subtypes were identified in each population using multiple marker genes (**Figures 1A, C**). The distribution and proportion of myeloid cell subtypes at different time points are shown in **Figure 1D** and **Figure 1E**. Macrophages increased rapidly in the first three days but decreased in the subacute and chronic phases. Microglia were the only myeloid cells in the sham group and were the major cell type in the subacute and chronic phases, consistent with previous studies (25).

### Unique marker genes and signatures of macrophages and microglia at different time points

**Figure 2:**
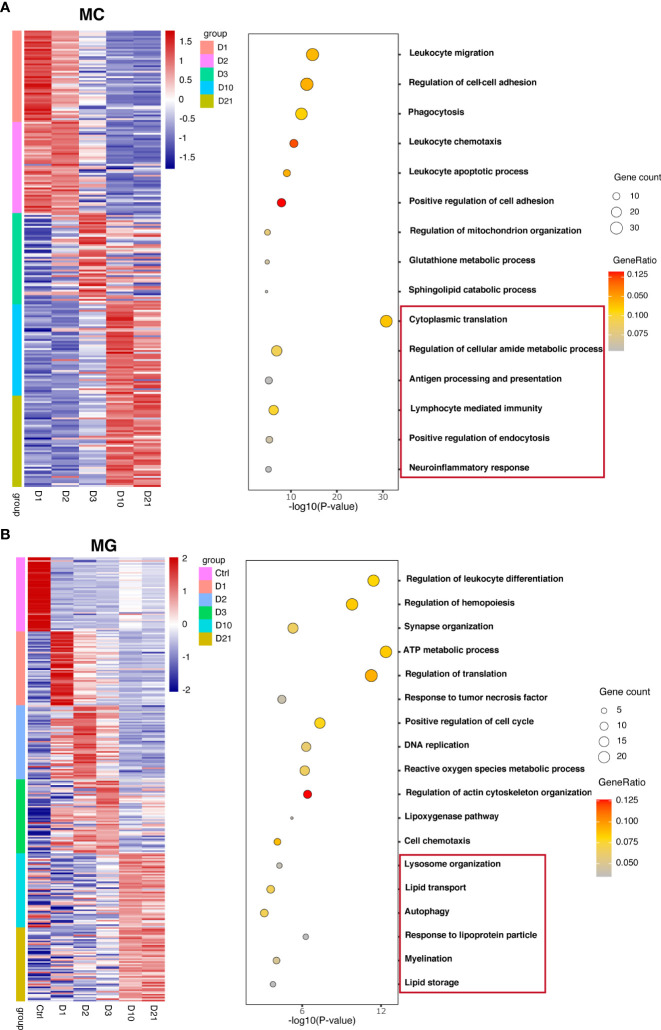
Unique marker genes and related gene ontology (GO) of biological processes in macrophages **(A)** and microglia **(B)** collected at different time points.

To investigate the biological functions of macrophages and microglia at different time points, differential analysis was conducted based on the AUC classifier and GO analysis. Macrophages and microglia had different DEGs and pathways 10 and 21 dpi compared with the first three days post injury but were indistinguishable between 10 dpi and 21 dpi. For example, GO terms specific to macrophages included cytoplasmic translation pathway, regulation of cellular amide metabolic processes, and antigen processing and presentation at 10 dpi, and lymphocyte-mediated immunity pathway, positive regulation of endocytosis, and neuroinflammatory response at 21 dpi (**Figure 2A**). GO terms specific to microglia included lysosome organization, lipid transport, and autophagy pathways at 10 dpi, and response to lipoprotein particles, myelination, and lipid storage pathways at 21 dpi (**Figure 2B**). Interestingly, the biological function of microglia indicates that microglia may produce lipid droplets and degrade them through autophagy to maintain lipid metabolism balance.

### APOE is the top upregulated gene in macrophages and microglia in the subacute and chronic phases

**Figure 3:**
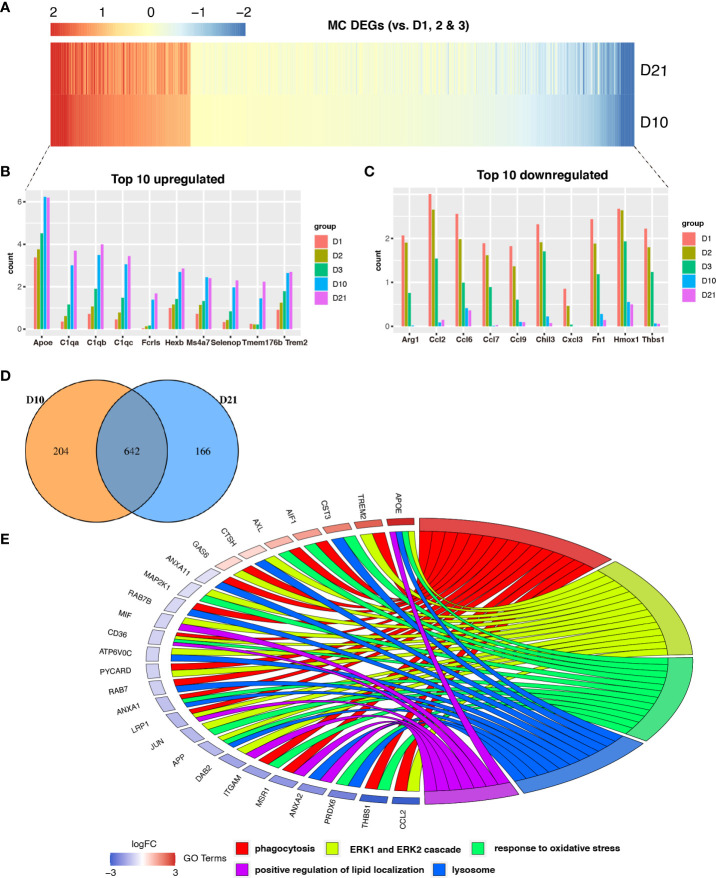
Analysis of differential gene expression in macrophages following spinal cord injury (SCI). **(A)** Heatmap showing differentially expressed genes (DEGs) between macrophages sampled 10 days post injury (dpi) and 21 dpi compared with macrophages sampled in the first three days. **(B, C)** Bar graph showing the top 10 upregulated and downregulated genes in macrophages sampled 10 dpi and 21 dpi. **(D)** Venn diagram showing an overlap between genes that were differentially expressed between macrophages sampled 10 dpi and those sampled 21 dpi. **(E)** Chord plot showing the gene ontology (GO) of biological processes associated with overlapping DEGs in macrophages sampled 10 dpi and 21 dpi.

We identified genes that were differentially expressed in macrophages during the subacute and chronic phases (**Figure 3A**). Compared to the acute phase, a total of 642 genes were differentially expressed in macrophages at 10 and 21 dpi (**Figure 3D**). The top 10 upregulated and downregulated DEGs are shown in the bar graphs in **Figure 3B** and **Figure 3C**, respectively. GO analysis showed that co-expressed genes, including *APOE*, *TREM2, CST3*, and *AIF1* were enriched for phagocytosis, ERK1 and ERK2 cascades, oxidative stress response, lysosomes, and positive regulation of lipid localization (**Figure 3E**). Notably, the TREM2-APOE pathway has been identified as a major regulator of dysfunctional microglia and as a therapeutic target for neurodegenerative diseases (26).

**Figure 4:**
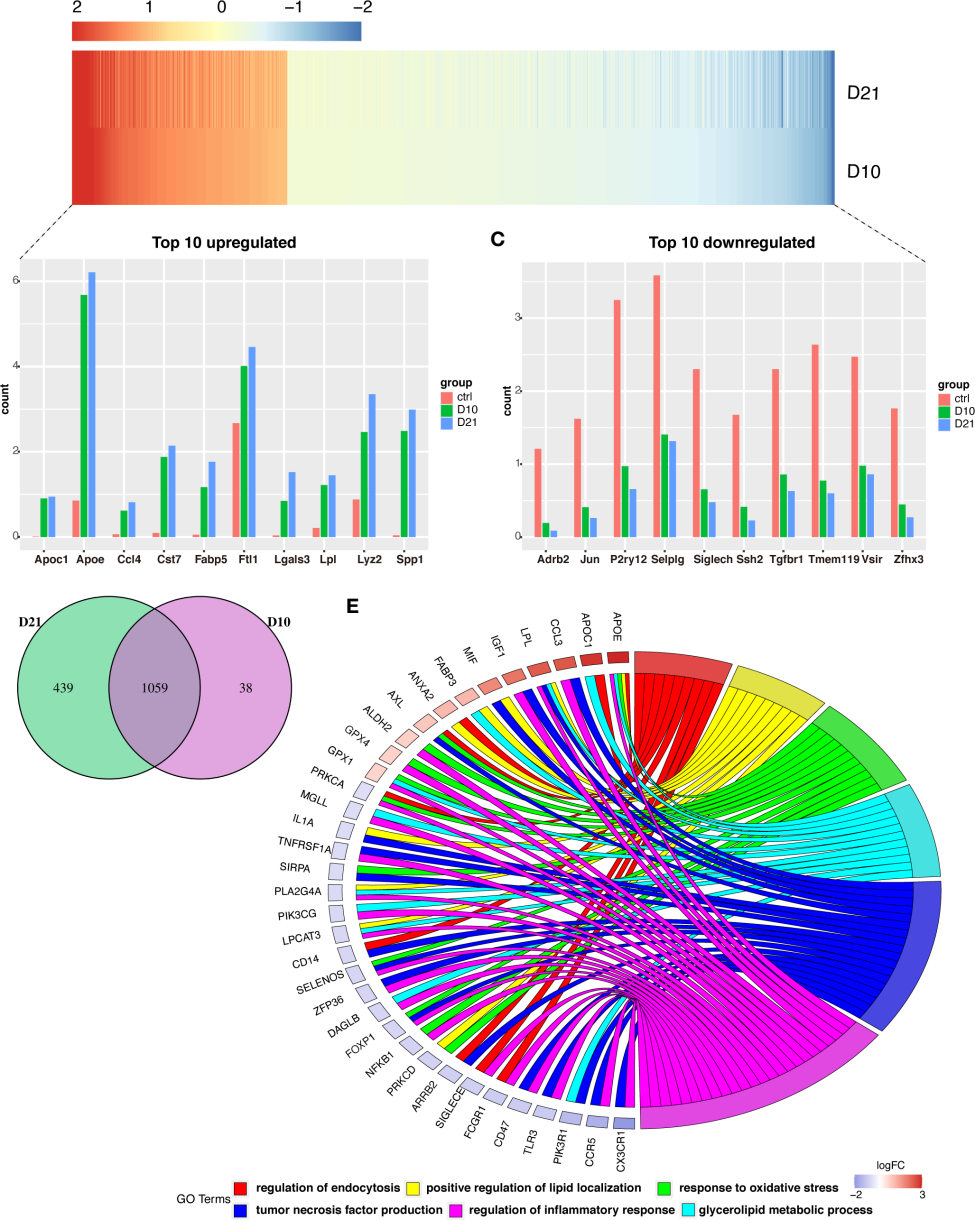
Analysis of differential gene expression in microglia following spinal cord injury (SCI). **(A)** Heatmap showing differentially expressed genes (DEGs) in microglia sampled 10- and 21-days post injury (dpi) compared with those sampled from the sham group. **(B, C)** Bar graph showing the top 10 upregulated and downregulated genes in microglia sampled 10 dpi and 21 dpi. **(D)** Venn diagram showing overlap between DEGs in microglia sampled 10 dpi and 21 dpi. **(E)** Chord plot showing the gene ontology (GO) of biological processes associated with overlapping DEGs in microglia sampled 10 dpi and 21 dpi.

A total of 1059 genes were differentially expressed at 10 and 21 dpi in microglia compared to the acute phase (**Figures 4A, D**). Of the top 10 upregulated genes (**Figure 4B**), the *FABP5* is thought to be neurotoxic as it promotes immune cell infiltration and the release of inflammatory factors (10). Additionally, LGALS3/Galectin-3 was recently identified as a critical factor in microglia-mediated neuroinflammation (27). Most of the top 10 downregulated genes were homeostatic microglial genes, including *JUN, P2RY12, SIGLECH, TMEM119*, and *TGFBR1* (**Figure 4C**). GO analysis showed that overlapping genes such as *APOE, APOC1, CCL3, LPL, IGF1, MIF*, and *FABP3* were involved in the regulation of phagocytosis, positive regulation of lipid localization, response to oxidative stress, production of tumor necrosis factor, regulation of inflammatory response, and metabolism of glycerolipids (**Figure 4E**).

### Temporal clustering analysis

**Figure 5:**
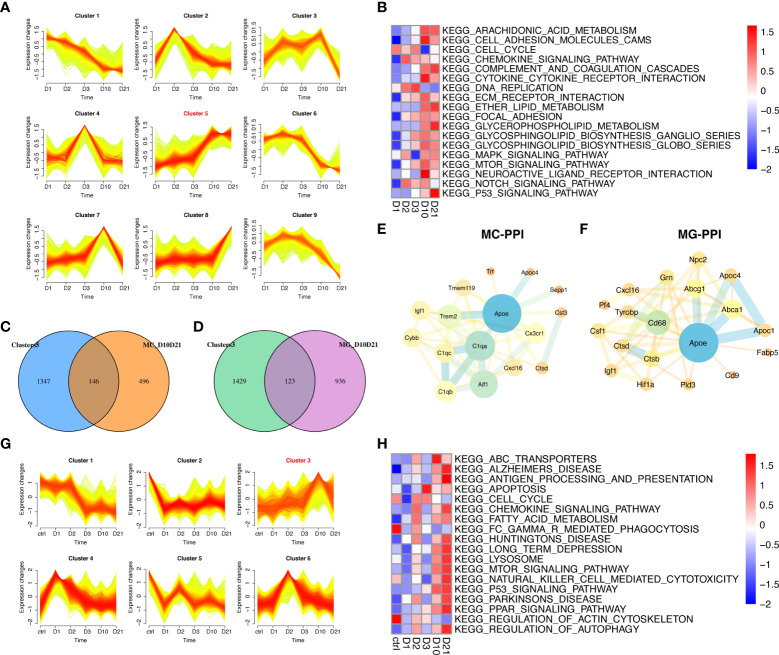
Temporal clustering analysis of macrophages and microglia following spinal cord injury (SCI). **(A)** Temporal clustering analysis of macrophages following SCI. **(B)** Heatmap showing gene set variation analysis (GSVA) of genes in cluster five and c2 KEGG gene sets. **(C)** Venn diagram showing an overlap between genes in cluster five and genes that were differentially expressed between macrophages sampled 10 days post injury (dpi) and those sampled 21 dpi. **(D)** Venn diagram showing an overlap between genes in cluster three and genes that were differentially expressed between microglia sampled 10 dpi and those sampled 21 dpi. **(E)** Protein-protein interaction (PPI) network showing an overlap of genes in cluster five and genes that were differentially expressed in macrophages sampled 10 dpi and those sampled 21 dpi. **(F)** PPI network showing the overlap between genes in cluster three and genes that were differentially expressed between microglia sampled 10 dpi and those sampled 21 dpi. **(G)** Temporal clustering analysis of microglia following SCI. **(H)** Heatmap showing GSVA of genes in cluster three and the c2 KEGG gene sets.

This study focused on the analysis of gene expression in macrophages and microglia during the subacute and chronic phases of SCI. Of the nine time-dependent expression patterns observed in macrophages, cluster five contained 1493 genes with upregulated expression at 10 dpi and 21 dpi (**Figure 5A**). GSVA showed that the enrichment scores of lipid metabolism-related pathways were higher at 10 dpi and 21 dpi, including arachidonic acid, ether lipid, and glycerophospholipid metabolism (**Figure 5B**). A total of 146 genes in cluster five overlapped with 642 genes that were differentially expressed between 10 dpi and 21 dpi (**Figure 5C**). PPI network analysis in macrophages identified APOE as a hub gene intersecting with DEGs such as *C1QA, AIF1, TREM2*, and *TMEM119* (**Figure 5E**). Six time-dependent expression patterns were identified in microglia (**Figure 5G**). Cluster three contained 1552 genes whose expression was upregulated at 10 and 21 dpi. GSVA showed that the pathways associated with neurodegenerative diseases, lysosomes, peroxisome proliferator-activated receptor (PPAR) signaling pathway, and autophagy had the highest scores at 21 dpi (**Figure 5H**). A total of 123 genes in cluster three overlapped with the 1059 genes that were differentially expressed at 10 dpi and 21 dpi in microglia (**Figure 5D**). PPI network analysis in microglia also identified APOE as a hub gene intersecting with DEGs such as *ABCA1, ABCG1, APOC1, CD68*, and *CTSB* (**Figure 5F**). These results demonstrate the essential role of APOE in macrophages and microglia during the subacute and chronic phases following SCI.

### Accumulation of lipid droplets in macrophages and microglia following SCI

**Figure 6:**
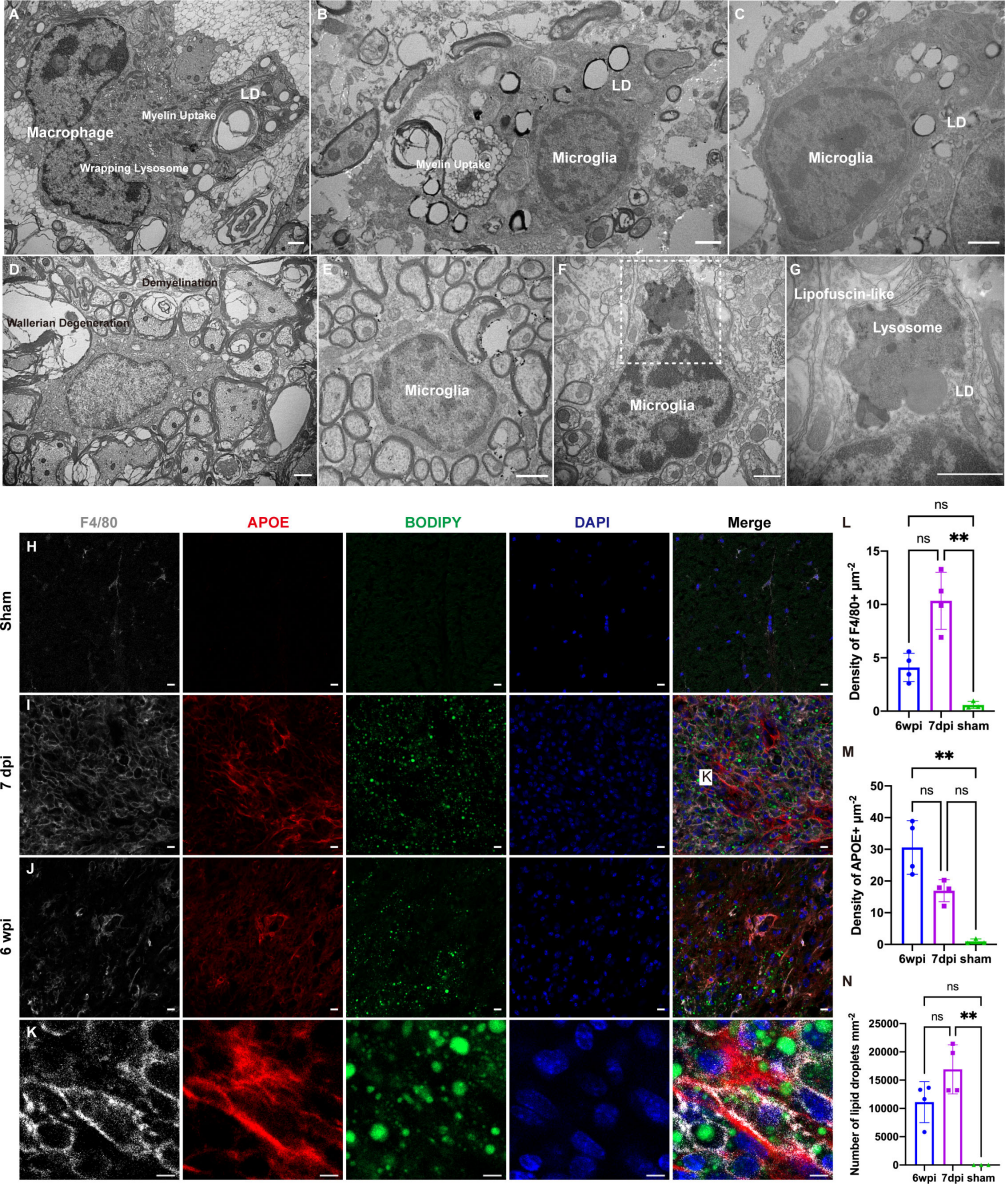
Transmission electron microscopy (TEM) analysis and immunofluorescence staining of a mouse model of cervical spinal cord hemi-contusion. **(A)** TEM image of macrophages sampled seven days post injury (dpi) showing myelin uptake, lipid droplet accumulation, and “wrapping lysosomes”. **(B, C)** Representative images of microglia sampled seven dpi showing myelin uptake, lipid droplet accumulation, and “wrapping lysosomes”. **(D)** TEM image of lesions sampled six weeks post injury (wpi) showing Wallerian degeneration and demyelination. **(E)** Representative image of healthy microglia in the sham group. **(F, G)** TEM images of microglia sampled six wpi showing lipofuscin granules and lipid droplets. The boxed area is shown in **(G)** at higher magnification. **(H–J)** Immunofluorescence staining of spinal cord samples showing F4/80+ microglia/macrophages (white), APOE (red), BODIPY (green) and DAPI (blue). Box is the approximate area where **(K)** was imaged. **(K)** High-magnification representative images of the APOE+ macrophages/microglia containing BODIPY+ lipid droplets. **(L)** Quantitation of F4/80+ densities showed that F4/80+ densities were significantly increased at 7dpi. **(M)** Quantitation of APOE+ densities showed that APOE+ densities were significantly increased at 6 wpi. **(N)** Quantitation of lipid droplets showed that the number of lipid droplets were significantly increased at 7dpi. LD, lipid droplets. ^ns^P > 0.05, **P < 0.05. Scale bar **(A–G)** = 1 µm. Scale bar **(H–J)** = 10 µm. Scale bar **(K)** = 5 µm.

To explore the reason for the transcriptional changes in macrophages and microglia after SCI, we analyzed the changes in the ultrastructure of a mouse model of spinal cord contusion injury using TEM. Healthy microglia had a small body, thin cytoplasm, and bean-shaped nuclei containing heterochromatin (**Figure 6E**) (28). Seven days after SCI, both macrophages and microglia contained myelin debris, had increased levels of lipid droplets and lysosomes, and the “wrapping lysosomes” engulfing the lipid droplets were similar to macrophage foam cells (**Figures 6A–C**) (29). Demyelination is a pathological hallmark of preclinical models of SCI (2). Six weeks after SCI, prominent Wallerian degeneration and chronic demyelination were observed in the lesions (**Figure 6D**). Similar to the lipid droplet-accumulating microglia in the aging brain (30), the microglia contained lipofuscin granules and lipid droplets (**Figures 6F, G**). It is difficult to distinguish macrophages from microglia, since they share several markers, such as F4/80 and iba-1. Immunofluorescence staining showed upregulated APOE expression with abundant lipid droplets in F4/80+ microglia/macrophages (**Figures 6H–N**).

### Deletion of *APOE* aggravates neuroinflammation and reduces recovery

**Figure 7:**
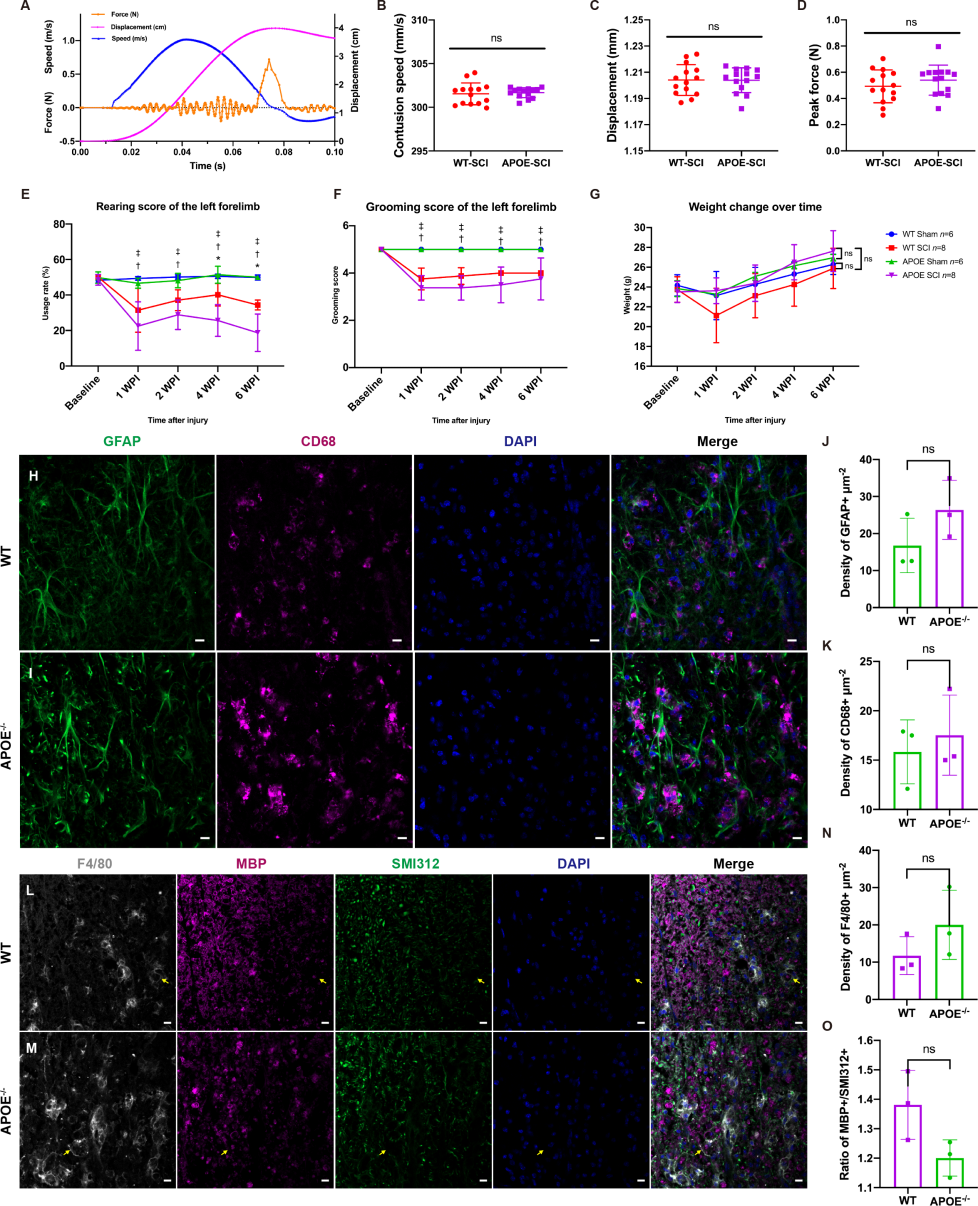
Apolipoprotein E (APOE) knockout aggravated dysfunction, increased neuroinflammation, and exacerbated white matter loss following spinal cord injury (SCI). **(A)** Typical changes in biomechanical parameters during contusion. Orange, contusion force; magenta, contusion displacement; blue, contusion speed. **(B–D)** There were no significant between-group differences in contusion displacement, speed or peak force. **(E)** Cylinder rearing test showed worse motor functional recovery in APOE^-/-^ mice following SCI. **(F, G)** There were no significant differences in grooming scores and body weight between the two injured groups. **(H–K)** APOE^-/-^ mice displayed aggravated neuroinflammation in the lesion rim. Green, GFAP+ astrocytes; purple, CD68+ macrophages/microglia; blue, DAPI+ cell nuclei. **(L-O)** APOE^-/-^ mice showed increased white matter loss and microglia/macrophage infiltration following SCI. The myelin debris was engulfed by microglia/macrophages (yellow arrows). White, F4/80; purple, myelin basic protein (MBP); green, SMI312+ axons; blue, DAPI. ^ns^P > 0.05, *P < 0.05 between the APOE-SCI and wild-type (WT)-SCI groups; ^†^P < 0.05 between the APOE-SCI and APOE-Sham groups; ^‡^P < 0.05 between the WT-SCI and WT-Sham groups. Scale bar **(H, I, L, M)** = 10 µm..

To investigate the function of APOE, we performed spinal cord contusion injury in APOE^-/-^ mice. Typical changes in the biomechanical parameters are shown in **Figure 7A**. There were no significant between-group differences in contusion displacement, speed, or peak force (**Figures 7B–D**). The cylinder rearing test on the ipsilateral forelimb revealed worse motor functional recovery in APOE^-/-^ mice, four and six weeks post-SCI (**Figure 7E**). Both groups showed similar ipsilateral grooming scores and changes in body weight post-SCI (**Figures 7F, G**). The reduction in the rate of utilization of the ipsilateral forelimb in APOE^-/-^ mice indicated the benefits of APOE in fostering recovery following SCI. Neuroinfiammation is a hallmark of SCI (31). Six weeks after SCI, however, APOE^-/-^ mice displayed aggravated neuroinflammation in the lesion rim (**Figures 7H–K**). Furthermore, APOE^-/-^ mice showed increased F4/80+ microglia/macrophage infiltration and white matter loss (**Figures 7L–O**). Although non-significant, these results were worthy of further investigation in subsequent studies.

### Myelin uptake in astrocytes and lysosome accumulation in macrophages and microglia in APOE^-/-^ mice

**Figure 8:**
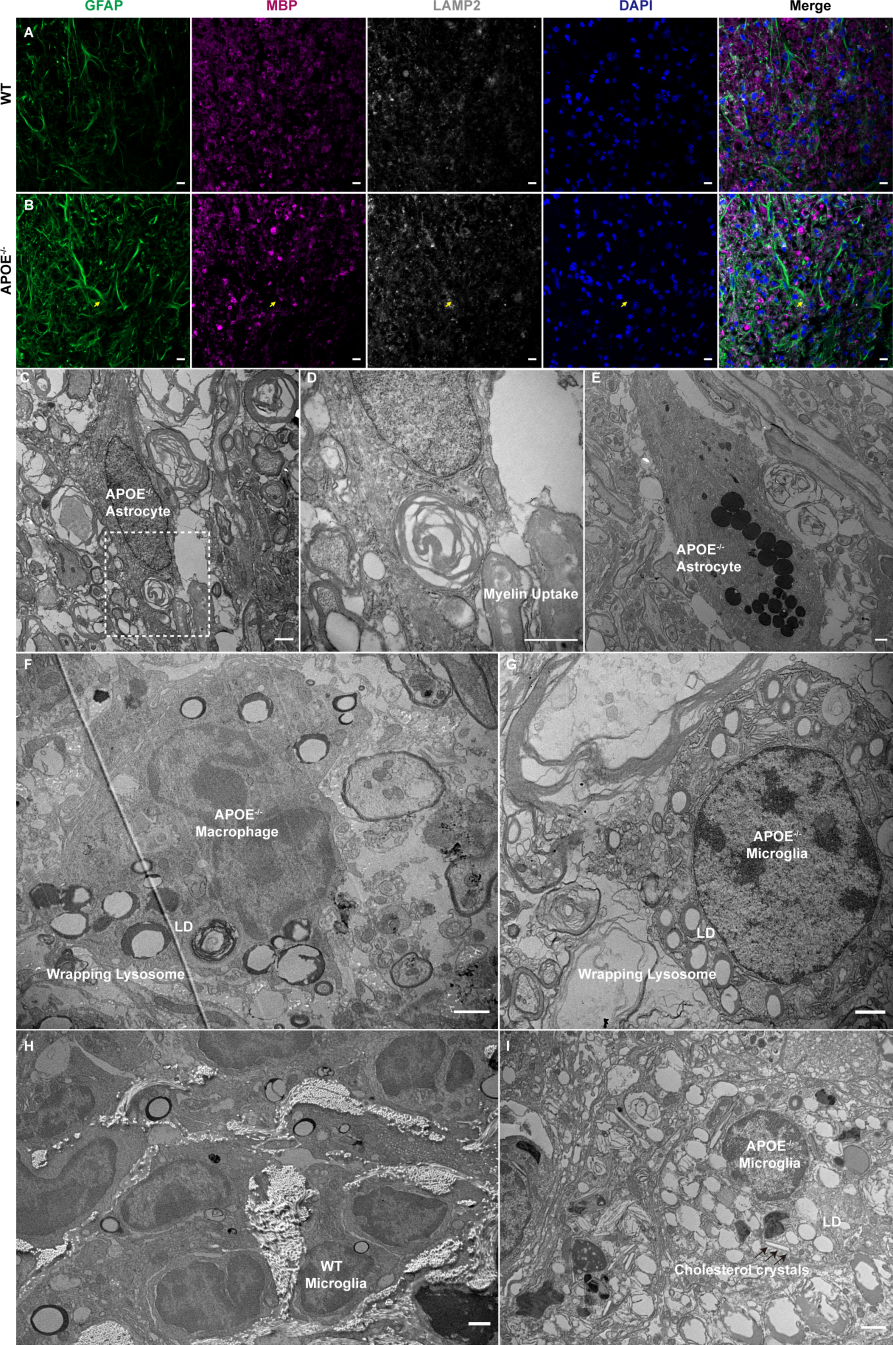
Myelin uptake in astrocytes and lipid droplets and lysosome accumulation in macrophages and microglia in APOE^-/-^ mice following spinal cord injury (SCI). **(A, B)** Myelin debris was engulfed by astrocytes in the lesion rim of APOE^-/-^ mice six weeks post injury (wpi) (yellow arrows). Green, GFAP; purple, MBP; white, LAMP2; blue, DAPI. **(C, D)** Transmission electron microscopy (TEM) images of astrocytes in APOE^-/-^ mice six wpi confirmed that the hypertrophic astrocytes contained degraded myelin debris. The boxed area is shown in **(D)** at higher magnification. **(E)** TEM image of astrocytes in APOE^-/-^ mice six wpi shows markedly elevated lysosomes. **(F, G)** TEM image of macrophages and microglia in APOE^-/-^ mice taken seven days post injury (dpi) shows increased number of lipid droplets and dense lysosome material. **(H)** Representative image of microglia in wild-type (WT) mice 16 wpi. **(I)** TEM image of microglia in APOE ^-/-^ mice 16 wpi shows markedly elevated lipid droplets and the formation of needle-like cholesterol crystals (black arrows). LD, lipid droplets. Scale bar **(A, B)** = 10 µm. Scale bar **(C–I)** = 1 µm.

Astrocytes phagocytose myelin debris and recruit immune cells during acute demyelination of brain tissue (32). We observed MBP in GFAP-positive cells in lesion rims of APOE^-/-^ mice (**Figures 8A, B**). TEM confirmed that the hypertrophic astrocytes contained degraded myelin debris (**Figures 8C, D**) and lysosomes accumulated in astrocytes (**Figure 8E**). Furthermore, APOE^-/-^ mice had increased numbers of lipid droplets and dense lysosomal material in macrophages and microglia (**Figures 8F, G**), suggesting that more lysosomes are activated in APOE^-/-^ mice following SCI. Interestingly, the TEM images of lesions sampled 16 weeks post injury showed the formation of needle-like cholesterol crystals (**Figures 8H, I**), which was similarly observed in the aged central nervous system (33). This confirmed chronic accumulation of cholesterol in microglia in APOE^-/-^ mice following SCI.

## Discussion

We identified APOE as a hub gene in both macrophages and microglia during the subacute and chronic phases of SCI. Histopathological analysis revealed the accumulation of lipid droplets and lysosomes in both macrophages and microglia. Furthermore, APOE^-/-^ mice showed worse functional recovery associated with increased neuroinflammation and white matter loss. APOE is the major cholesterol carrier involved in cellular lipid efflux in the central nervous system (33). Our study indicates that APOE and the associated cholesterol efflux might be therapeutically targeted to promote recovery following SCI.

Myelin and cellular debris are mainly cleared by monocyte−derived macrophages and resident microglia, creating a pro-regenerative environment (5). Macrophages are attracted to the injury site at around 3 days after SCI and reach a peak at 7 days after SCI (16). Macrophages play an important role in debris clearance, but excessive myelin debris uptake leads to the formation of “foamy macrophages” and subsequent death (6, 15). The primary injury causes glial necrosis within the lesion epicenter. Microglia are rapidly activated and proliferate after SCI (34). The efficiency of myelin phagocytosis and proliferation rate of microglia is much higher than those of macrophages (5). Therefore, microglia were the major myeloid cell type in the subacute and chronic phases of SCI. We found that microglial marker genes expressed 10 and 21 dpi were associated with lipid transport, autophagy, and lipid storage. Furthermore, autophagy regulation and the PPAR signaling pathway had high enrichment scores in microglia sampled at 21 dpi. Lipid droplets are organelles that store lipids and play a central role in cellular metabolism and lipid homeostasis (35). Lipid droplets have variable protein and lipid composition and their size, and the number of lipid droplets, are regulated *via* autophagy (36). PPAR-γ can upregulate the expression of *ABCA1* and *ABCG1* to boost lipid efflux (5). Stimulating the autophagy of lipid droplets and promoting lipid efflux in macrophage foam cells is an attractive therapeutic strategy for atherosclerosis (29). Thus, promoting macrophage autophagy and lipid efflux may reduce secondary damage in SCI.

Upregulated APOE expression in microglia is common during development, damage, and disease (37). In this study, we found that APOE is involved in multiple pathways and interacts with multiple genes in macrophages and microglia. The different effects of APOE depend on its cellular origin, binding lipid molecules, and microenvironment (38). APOE^-/-^ mice showed impaired remyelination and increased phagocyte infiltration in a demyelination model (33). We found that APOE^-/-^ mice showed worse functional recovery following SCI, consistent with previous reports (39, 40). The uptake of myelin debris by macrophages and microglia was confirmed using TEM. Myelin has a high lipid composition, particularly cholesterol (41). Our results suggest that cholesterol overload in macrophages and microglia may induce a maladaptive immune response that aggravates secondary damage. Further studies are required to explore the stimulation of reverse cholesterol transport.

Traumatic SCI leads to progressive cord atrophy and neurodegeneration (42). We observed clear demyelination and Wallerian degeneration on the ipsilateral side of the injury epicenter six weeks post injury (wpi) using TEM. Furthermore, we identified lipofuscin granules and lipid droplets in the microglia 6 wpi. Lipid droplet-rich microglia have recently been implicated in the release of large quantities of reactive oxygen species and proinflammatory cytokines in the aging brain, and nearly half of the constituents of lipid droplets are glycerolipids, in line with the transcriptomics data (10), although few of these are cholesteryl esters (30). Likewise, lipid droplets accumulate in microglia in patients with Alzheimer’s and Parkinson’s diseases (43, 44). Interestingly, the number and morphology of lipid droplets differed in microglia sampled seven dpi and six wpi. The effects of lipid droplets on microglia may depend on their composition, which is affect by different environmental conditions (30). It will be worth analyzing the content of lipid droplets in macrophages and microglia collected at different time points following SCI.

This study has several limitations. First, the numbers of samples and cells collected 21 dpi were small and no macrophages were present in samples used in the sham operation as expected, potentially introducing bias. Second, the cause of transcriptional changes and the effects of APOE were validated and explored using wild-type and APOE^-/-^ spinal cord contusion injury mouse models. Although we did not use exogenous APOE treatment or other methods to increase cholesterol efflux in the present study, previous *in vivo* study using COG112, an APOE mimetic peptide combined a protein transduction domain antennapedia to improve blood-brain barrier and cell membrane penetration, suggest neuroprotective roles of endogenous APOE in reducing neuroinflammation and white matter loss after SCI (40). Third, the APOE^-/-^ knockout is not myeloid-specific, and the molecular mechanisms underlying the phenotypes of APOE^-/-^ mice have not been fully elucidated. Since APOE has multiple functions in different environmental conditions (38), high-throughput assays can be used to elucidate differences in molecular mechanisms between wild-type and APOE^-/-^ mice following SCI.

Taken together, our study demonstrated that APOE is a hub gene in both macrophages and microglia in subacute and chronic phases of SCI. APOE knockout aggravates neurological dysfunction, increases neuroinflammation, and exacerbates white matter loss. Targeting APOE and related cholesterol efflux may be a promising strategy for reducing neuroinflammation and promoting recovery following SCI.

## Data availability statement

The datasets presented in this study can be found in online repositories. The names of the repository/repositories and accession number(s) can be found in the article/supplementary material.

## Ethics statement

The animal study was reviewed and approved by Laboratory Animal Care and Use Committee of Nanfang Hospital, Southern Medical University.

## Author contributions

X-QY, J-YC, Q-AZ, and J-TC conceived and designed the research. X-QY, Z-HY, Z-CH, Y-QZ, Z-PH, K-WT, and J-HL performed the experiments. RH collected the transcriptomics dataset under the supervision of SP and performed the pre-processing analysis. Y-ML and Z-TZ helped with the transmission electron microscopy analyses. X-QY, J-YC, Z-HY, and K-WT analyzed the data. X-QY, J-YC, and J-TC wrote the paper. All authors contributed to the article and approved the submitted version.

## Funding

J-TC was supported by National Natural Science Foundation of China (82172492), and the Science and Technology Planning Project of Guangdong Province, China (2017B010110012). Z-CH was supported by National Natural Science Foundation of China (81902217). J-HL was supported by Guangzhou Science and Technology Plan Project (202102021244).

## Acknowledgments

We thank Y-ML and Z-TZ for excellent technical assistance with transmission electron microscopy analyses. We thank Dr. Jianming Zeng (University of Macau), and all the members of his bioinformatics team, biotrainee, for generously sharing their experience and codes.

## Conflict of interest

The authors declare that the research was conducted in the absence of any commercial or financial relationships that could be construed as a potential conflict of interest.

## Publisher’s note

All claims expressed in this article are solely those of the authors and do not necessarily represent those of their affiliated organizations, or those of the publisher, the editors and the reviewers. Any product that may be evaluated in this article, or claim that may be made by its manufacturer, is not guaranteed or endorsed by the publisher.

## Abbreviations

SCI, spinal cord injury; APOE, apolipoprotein E; DPI, days post injury; WPI, weeks post injury; WT, wild-type; scRNA, single-cell RNA; DEGs, differentially expressed genes; GEO, gene expression omnibus; UMAP, uniform manifold approximation and projection; AUC, under the curve; GO, gene ontology; GSVA, gene set variation analysis; KEGG, Kyoto Encyclopedia of Genes and Genomes; PPI, protein-protein interaction; PBS, phosphate-buffered saline; TEM, transmission electron microscopy; PPAR, peroxisome proliferator-activated receptor; MBP, myelin basic protein.
